# Design of a Highly Effective Therapeutic HPV16 E6/E7-Specific DNA Vaccine: Optimization by Different Ways of Sequence Rearrangements (Shuffling)

**DOI:** 10.1371/journal.pone.0113461

**Published:** 2014-11-25

**Authors:** Fahad N. Almajhdi, Tilo Senger, Haitham M. Amer, Lutz Gissmann, Peter Öhlschläger

**Affiliations:** 1 Department of Botany and Microbiology, College of Science, King Saud University, Riyadh, Saudi Arabia; 2 German Cancer Research Center, Heidelberg, Germany; 3 Virology Department, Faculty of Veterinary Medicine, Cairo University, Giza, Egypt; 4 Department of Chemistry and Biotechnology, Aachen University of Applied Sciences, Jülich, Germany; University of Massachusetts Medical Center, United States of America

## Abstract

Persistent infection with the high-risk Human Papillomavirus type 16 (HPV 16) is the causative event for the development of cervical cancer and other malignant tumors of the anogenital tract and of the head and neck. Despite many attempts to develop therapeutic vaccines no candidate has entered late clinical trials. An interesting approach is a DNA based vaccine encompassing the nucleotide sequence of the E6 and E7 viral oncoproteins. Because both proteins are consistently expressed in HPV infected cells they represent excellent targets for immune therapy. Here we report the development of 8 DNA vaccine candidates consisting of differently rearranged HPV-16 E6 and E7 sequences within one molecule providing all naturally occurring epitopes but supposedly lacking transforming activity. The HPV sequences were fused to the J-domain and the SV40 enhancer in order to increase immune responses. We demonstrate that one out of the 8 vaccine candidates induces very strong cellular E6- and E7- specific cellular immune responses in mice and, as shown in regression experiments, efficiently controls growth of HPV 16 positive syngeneic tumors. This data demonstrates the potential of this vaccine candidate to control persistent HPV 16 infection that may lead to malignant disease. It also suggests that different sequence rearrangements influence the immunogenecity by an as yet unknown mechanism.

## Introduction

Papillomaviruses comprise a large group of small DNA viruses with a very distinct biology. Being restricted to skin and mucosa without a viremic phase during virus replication, the pattern of their gene expression is tightly linked to the differentiation of the epithelium. The highest load of antigens appears in the keratinized upper cell layers where synthesis of the structural proteins and assembly of particles takes place. Virus maturation *per se* does not cause cells death. Instead the viral E4 protein facilitates particle release from the desquamating cells by disrupting the intermediate filaments of the keratinocyte cytoskeleton. This strategy of low profile enables papillomaviruses to bypass the surveillance of the immune system and hence to persist for different periods of time within the affected epithelial site.

Persistence is a particular hallmark of the so-called high-risk human papillomaviruses (HPV) possibly since they replicate only in a few cells within a lesion [1], [2], hence they may not be able to maintain themselves in the human population if they only have the chance for a one-burst replication. Molecularly the state of persistence is not understood yet we know from a large number of cohort studies that persistence is the precondition for progression of a benign cervical lesion (LSIL) towards more malignant disease, i.e. high squamous intraepithelial lesion (HSIL) and ultimately cancer. Follow up of persistently infected women without clinical signs carry a risk for an abnormal Pap smear of about 25% within the next 12 years [3].

HPV 16 and HPV 18 are the most important types for the development of cervical cancer and other malignant tumors of the anogenital tract and of the head and neck. A few years ago two vaccines against these viruses became commercially available. Clinical trials and the first reports after launching vaccination campaigns in countries such as Australia and the UK demonstrated highly efficient protection against persistent infection and precancerous lesions [4], [5], [6], [7], [8]. Data on the influence on cancer incidence are expected to arise 15–20 years after initiation of mass immunization.

From the clinical trials, it also became evident that the current vaccines have no therapeutic activity, i.e. they are unable to eliminate existing infections [9]. On the other hand, studies on the natural history of cervical dysplasia strongly suggest a role of cellular immune responses directed against the viral proteins E2, E6 and/or E7 in controlling persistent infections and progression towards high-grade lesions. In the past, vaccine candidates in various formulations (fusion proteins, peptides, minigenes, etc) targeting these viral proteins have been evaluated in numerous preclinical studies in mice (for summary see [10]). Some of these candidates moved forward into early clinical trials aiming at safety and immunogenicity as primary endpoints and possibly providing some clues for efficacy. For various reasons (discussed by [11]), the studies – although sometimes yielding promising results – did not advance into further investigation. Proof of principle for the concept of HPV-specific immune therapy was obtained in two trials where women with therapy-resistant HPV 16 positive high-grade vulval intraepithelial neoplasia (VIN 3) were immunized with an HPV 16 E6/E7/L2 fusion protein or long overlapping peptides encompassing the HPV 16 E6 and E7 proteins. At 12 months of follow-up the authors noted clinical responses in 63% (12 of 19 patients) or 79% (15 of 19 patients), respectively [12], [13]. These results were achieved by the aid of potent adjuvants, which lead to considerable local side effects. These events might be acceptable given the severity of premalignant vulval disease but will not be tolerated when women with persistent infection without clinical symptoms are to be treated.

An attractive alternative to application of proteins or peptides is genetic immunization either as naked DNA, by transfer through a viral vector or as a combination of both in a heterologous prime-boost scheme. For the use of the viral oncoproteins E6 and E7 as immunogen, we have developed the concept of gene shuffling to deprive them of their transforming yet to maintain all possible T cell epitopes [14], [15]. Here we describe the generation of HPV 16 genes containing shuffled segments of E6 and E7 in one molecule. Out of 8 candidates we identified one with superior immunogenicity which has the potential of clinical development.

## Materials and Methods

### Generation of shuffled genes

**Figure 1:**
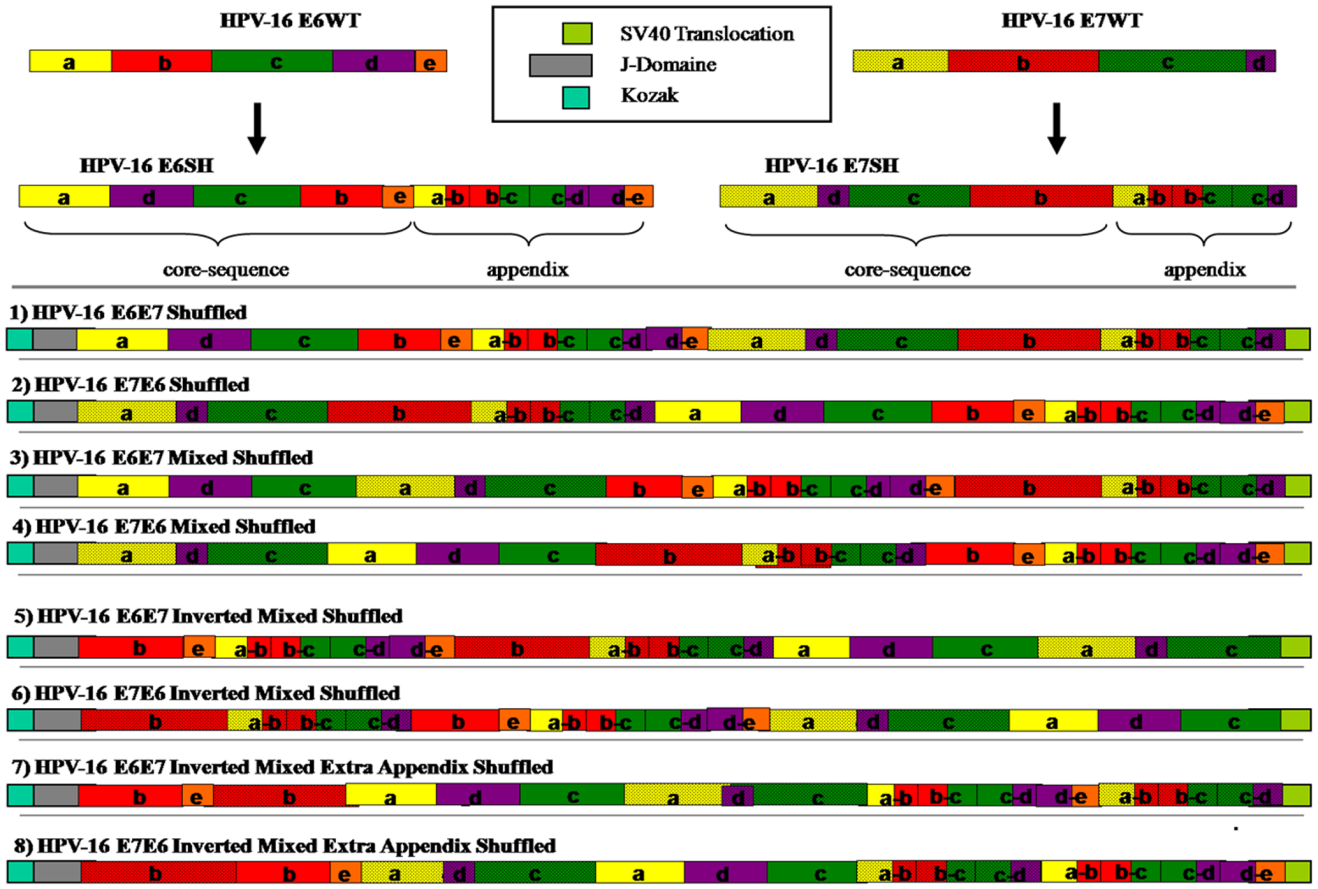
Representation of the different versions of HPV-16 E6/E7 shuffled genes. HPV-16 E6 and E7 wild-type protein genes were dissected at the transformation sites (see Materials and Methods S1) and rearranged to disrupt their activities. To avoid the loss of T-cell epitopes, the original sequences were added as appendices. Eight constructs with combined E6 and E7 shuffled genes were designed as shown. Kozak consensus sequence and J-Domain were added at the 5′-end and SV40 translocation sequence was added at the 3′-end.

Eight different versions of the HPV-16 E6/E7 shuffled genes were generated by assembly of synthetic oligonucleotides (Figure 1). E6 and E7 wild-type protein genes were dissected to disrupt the transformation-relevant sequences at nucleotide positions 114, 213, 333 and 432 of E6 and 72, 177 and 276 of E7. The five segments of E6 (a-b-c-d-e) were rearranged in the order (a-d-c-b-e), while the four segments of E7 (a-b-c-d) were rearranged in the order (a-d-c-b). Since shuffling could affect the putative T-cell epitopes located at the disruptive sequences, the original junction sequences were added as appendices at the end of core sequences.

The artificial genes were synthesized and codon-optimized for the human system by GENEART (Regensburg, Germany) and cloned via 5′-*Hind*III and 3′-*Xba*I into the immunization vector pTHkan [16], which has already been used in a version without any antibiotic resistance gene (pTHr) in humans [17]. The Kozak sequence [18] and the J-domain [19] were attached on the 5′-end. The SV40 enhancer sequence [20] was attached on the 3′-end of the genes, respectively. The composition of the 8 artificial genes is shown schematically in Materials and Methods S1.

### Tumor cell culture

**RMA-S cells** [21] were cultured in RPMI 1640 supplemented with heat-inactivated 5% (v/v) fetal calf serum (FCS, Gibco, Eggenstein, Germany), 2 mM L-glutamine, penicillin (100 U/ml) and streptomycin (100 µg/ml). **C3 cells** derived from embryonic mouse cells transfected with the complete HPV-16 genome [22] were cultured in RPMI 1640 supplemented with heat-inactivated 5% (v/v) FCS, 2 mM L-glutamine, penicillin (100 U/ml), streptomycin (100 µg/ml) and kanamycin (0.1 mg/ml). **293tt cells** [23] were cultured in DMEM supplemented with10% (v/v) FCS, 2 mM L-glutamine, penicillin (100 U/ml), streptomycin (100 µg/ml), and amphotericin B (0.25 µg/ml) (Sigma, St. Louis, MO).

### Immunization of mice

Six-to-eight week old female C57BL/6 mice (owner bred) were kept under SPF isolation conditions and standard diet at the animal facilities of the German Cancer Research Center, Heidelberg, Germany. Agarose-gel verified plasmids (>95% supercoiled, QIAGEN EndoFree Plasmid Kit; preparations contained less than 0.1 endotoxin units/µg plasmid DNA as tested earlier by Limulus endotoxin assay) were injected. For CTL analysis, animals were immunized once (100 µg DNA/per animal [50 µg DNA in 50 µl PBS per *musculustibialis anterior* i.m.]).Ten to 12 days after vaccination, animals were sacrificed and spleens were isolated.

Two×10^7^ spleen cells (pretreated with ACT lysis buffer [17 mMTris/HCl, 160 mM NH_4_Cl, pH 7.2] to deplete erythrocytes) were used directly for Elispot-assays or co-cultured with 2×10^6^ irradiated (100 Gy) RMA-S cells loaded either with E6 or E7 peptide in 25 cm^2^ culture flasks in αMEM (Sigma, Deisenhofen, Germany) supplemented with 10% FCS, 0.1 mMβ-mercaptoethanol, 4 mM glutamine and antibiotics for 5–6 days at 37°C and 7.5% CO_2_ in a humidified incubator.

### *In vivo* tumor regression

C57BL/6 mice received 0.5×10^6^ HPV-16 E7 expressing C3 cells [22] in 100 µl of PBS, subcutaneously in the right flank (needles: 20G 1½″ BD Microlance 3). When small tumors were palpable in all animals (14–17 days after tumor cell injection, defined as “day 0”), the DNA vaccine was injected i.m twice on days 0 and 7 or 8 in both *musculustibialis anterior*, as described above. Tumor sizes were measured with a caliper. Mice were sacrificed when the tumor size reached 400 mm^2^ or when tumors were bleeding. Tumor sizes of the mice within a group were calculated as arithmetic means with standard deviation (SD). All operations on live animals were performed under Isofluraneanaesthesia.

All animal experiments were performed with approval by and in accordance with regulatory guidelines and standards set by the institutional review board at Regierungspraesidium, Karlsruhe, Germany (35-9185.81/G-48/06)

### Detection of T cells responses

**IFN-γ Elispot assays** were performed *ex vivo* as previously described [14]. The granzyme B Elispot assay was performed similarly to the IFN-γ Elispot Assay, the anti-mouse granzyme capture antibody (100 ng/well, AF1865; R&D Systems, Minneapolis, USA) and the biotinylated anti-mouse granzyme detection antibody (50 ng/well, BAF1865; R&D Systems, Minneapolis, USA) were used. Splenocytes were seeded in 2-fold serial dilutions from 200,000 to 25,000 cells per well. One row was left untreated (negative control), the second received 200 ng of pokeweed mitogen/well (Sigma, Deisenhofen, Germany) in 2 µl of PBS (positive control), whereas the third and fourth received 0.2 µmol of H2Db-restricted HPV-16 E749-57/E6 48-57peptide in 2 µl of PBS/well (test sample), respectively. Spots of the negative control (untreated) were subtracted from the spot number in the corresponding test sample.

The **^51^Cr-release assays** were performed after one *in vitro* restimulation of murine spleen cells. One×10^4^ Na_2_^51^CrO_4_labeled (0.05 mCi) target cells/well (unloaded RMA-S cells or RMA-S cells loaded either with E6 or E7 peptide) were incubated together with decreasing numbers of effector cells in 200 µl per well of a 96-well round bottom plate (Costar, Corning, USA) for 4 h. Subsequently, 50 µl of supernatant was harvested from each well and the released radioactivity was measured in a Microbeta counter (Wallac, Turku, Finland). Specific lysis was calculated according to the formula: percent specific lysis = [(cpm of the sample−spontaneous release)/(total release−spontaneous release)]×100, where total release and spontaneous release are measured in counts per minute (cpm). Spontaneous chromium release was determined by using ^51^Cr-labeled target cells without effector cells, and total chromium release was determined by adding 2% Triton X-100 to lyse the labeled target cells.

### Statistical analysis

Differences of means between experimental and control group were considered statistically significant when p was less than 0.05 by unpaired Students t-test.

## Results

### Generation and expression of the novel artificial HPV-16 shuffled genes

It was the aim of this study to generate a number of HPV 16 E6/E7 shuffled genes and to identify the construct with the highest immunogenicity for further development as a vaccine candidate. A total of 8 novel artificial HPV-16 E6 and E7 genes were generated as described in Materials and Methods S1. We took advantage of the SV40 enhancer sequence mediating an increase in gene transfer from the cytoplasm into the nucleus [24], [25]. Moreover, we fused the large T antigen-derived hsp73-binding Dna J-like domain to the 5′end of the genes aiming at a more effective MHC-I cross-presentation and cross-priming of CTLs [19] (Figure 1).

### Immunogenicity

**Figure 2:**
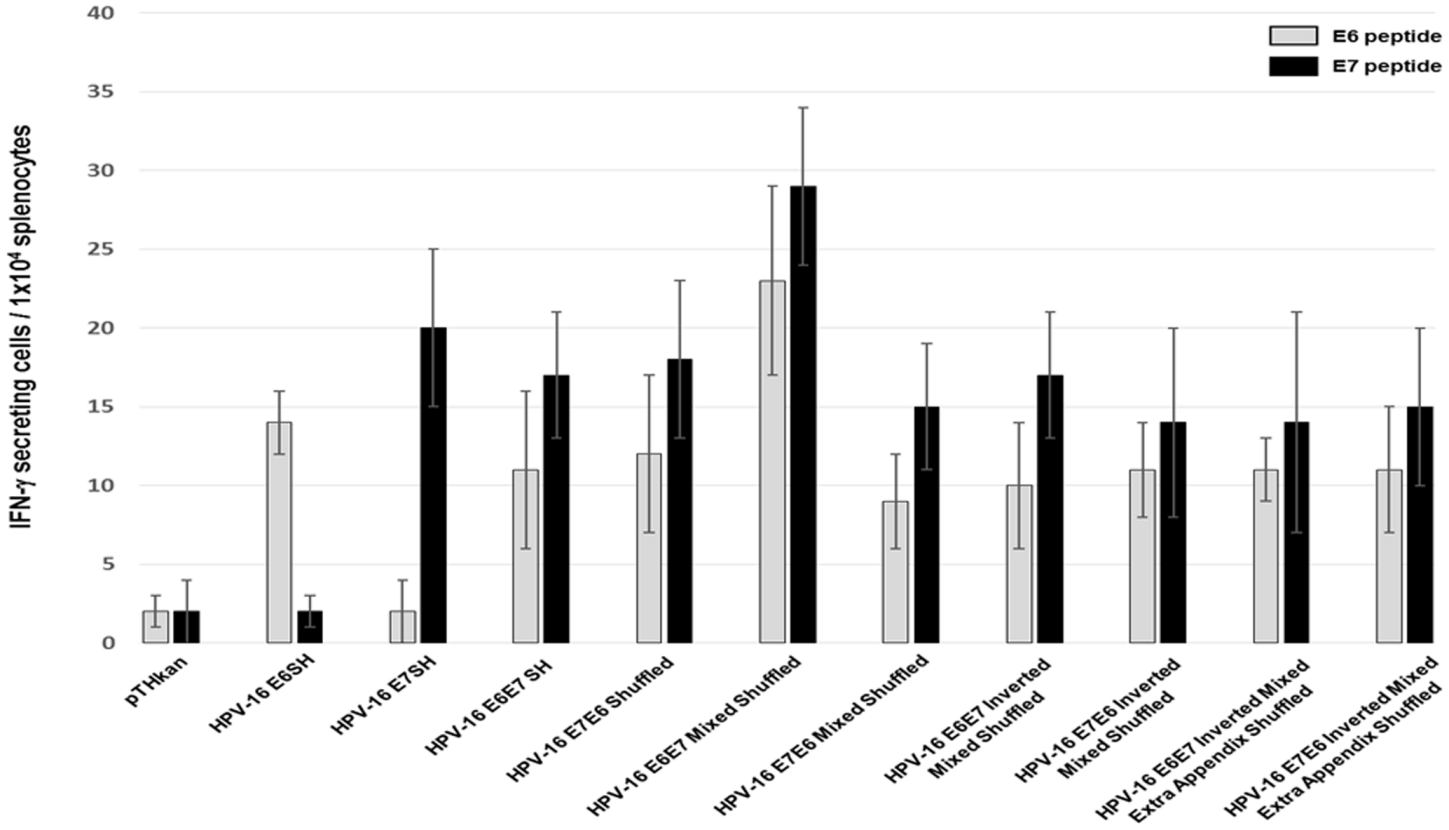
*Ex vivo* IFN-γ Elispot responses after DNA immunization. Four mice per group were immunized once i.m. with 100 µg empty vectors (pTHkan) or with 100 µg HPV-16-encoding vectors as indicated. The bars show the mean numbers of IFN-γ secreting cells/1×10^4^ splenocytes ± SD. One of two experiment yielding very similar results is shown.

We determined the immunogenicity of the eight novel gene constructs providing all possible occurring CD8 epitopes of the E6 and E7 genes of HPV-16. First, we measured IFN-gamma and granzyme B secretion of splenocytes *ex vivo*, respectively. As positive controls, we included the shuffled E6 and E7 genes (HPV-16 E6SH, HPV-16 E7SH), immunogenicity of which had been determined earlier [14], [26]. All eight investigated novel gene constructs induced robust IFN-gamma responses in comparison to the control (empty vector pTHkan; IFN-gamma secreting cells/1×10^4^ splenocytes: 2±1 after E6 peptide stimulation and 2±2 after E7 peptide stimulation; figure 2). Splenocytes of the HPV-16 E6E7 mixed shuffled immunized group revealed the highest E6- (23±6, *vs* control p: 0.0005) and E7-specific (29±5, *vs* control p<0.0001) IFN-gamma responses. The measured responses were also significantly higher compared to the animal group with the second strongest induction of the cytotoxic T lymphocytes (E6 stimulation: E6E7 mixed shuffled *vs* E6SH, p = 0.03; E7 stimulation: E6E7 mixed shuffled *vs* E7SH, p = 0.04).

**Figure 3:**
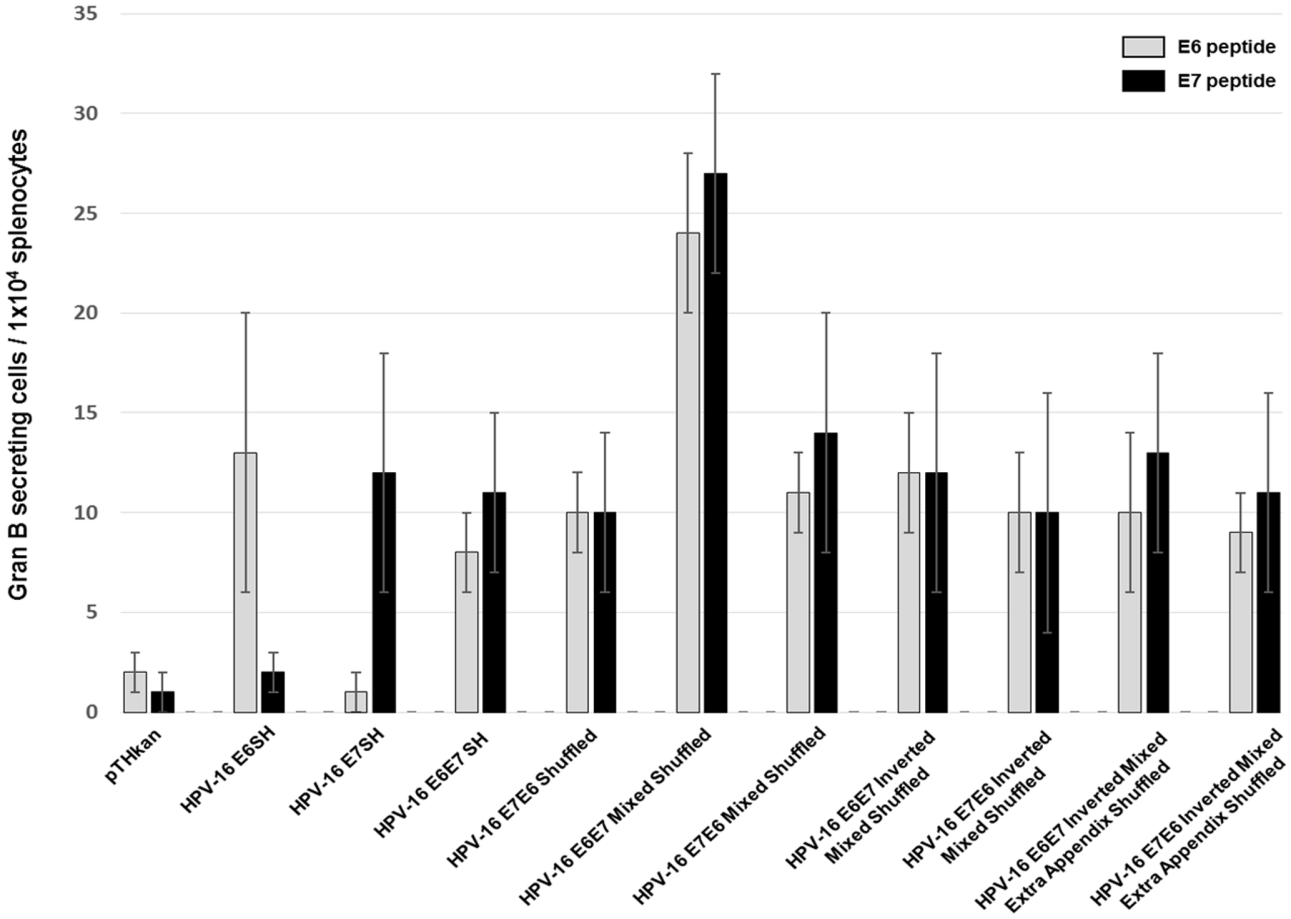
*Ex vivo* granzyme B Elispot responses after DNA immunization. Four mice per group were immunized once i.m. with 100 µg empty vectors (pTHkan) or with 100 µg HPV-16-encoding vectors as indicated. The bars show the mean no. of granzyme B secreting cells/1×10^4^ splenocytes ± SD. One of two experiment yielding very similar results is shown.

We obtained similar results in the granzyme B Elispot assays. Granzyme B is critical in the induction of apoptosis in target cells by cytotoxic T lymphocytes. Again, the HPV-16 E6E7 mixed shuffled construct induced the strongest activation of cytotoxic T lymphocytes (24±4 after E6 stimulation, 27±5 after E7 stimulation) (figure 3). The E6SH construct triggered the second strongest granzyme B secretion after E6 peptide stimulation (p = 0.03), whereas immunization with the artificial E7E6 mixed shuffled gene generated the second highest E7-specific response (p = 0.02).

**Figure 4:**
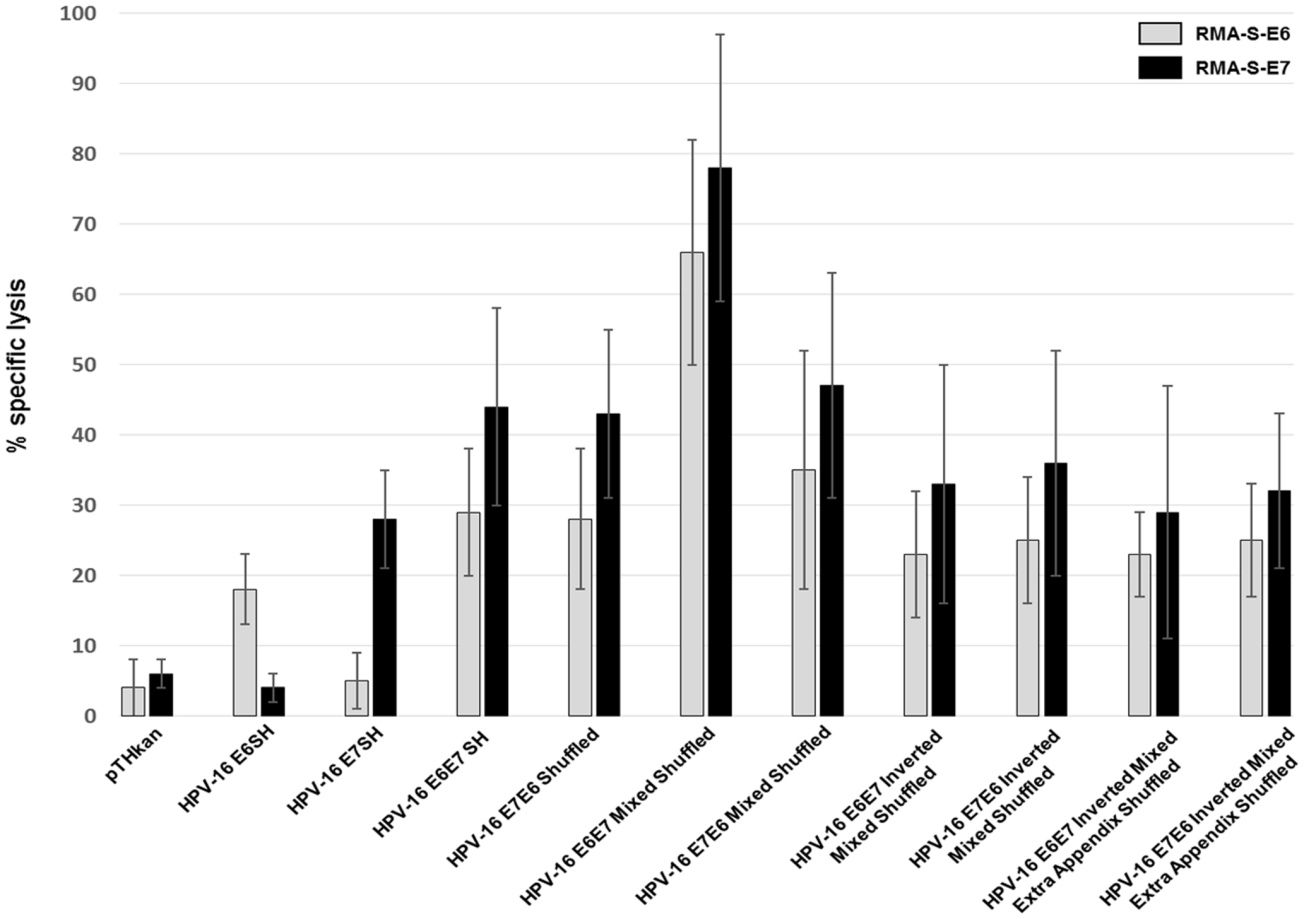
CTL activity against wildtype HPV-16 E6 and E7. Four mice per group were immunized once i.m. with 100 µg empty vectors (pTHkan) or with 100 µg HPV-16-encoding vectors as indicated and splenocytes were tested by ^51^Cr-release assays after one round of *in vitro* restimulation for lysis of RMA-S cells loaded with E6- or E7 peptide, respectively. Given are the mean of specific lysis in % ± SD. One of two experiment yielding very similar results is shown.

In order to characterize the cellular immune response in more detail, we decided to determine the cytotoxic potential of the T lymphocytes in^51^Cr-release assays after one round of *in vitro* restimulation of the splenocytes. Five to 6 days after *in vitro* restimulation, the splenocytes were co-incubated with ^51^Cr-labeled RMA-S target cells. Consistent with the Elispot assays, the splenocytes of the HPV-16 E6E7 mixed shuffled treated mice mediated the highest specific lysis of RMA-S cells loaded with E6- or E7-peptide, respectively (maximal specific lysis RMA-S-E6: 66%±16, RMA-S-E7: 78%±19) (figure 4). The second highest lysis of E6 or E7 loaded target cells was induced by the splenocytes of E7E6 mixed shuffled vaccinated mice, however the measured responses were significantly lower than in the E6E7 mixed shuffled immunized animals (RMA-S-E6, p = 0.03, RMA-S-E7, p = 0.04).

We concluded from the different experiments that the HPV-16 E6E7 mixed shuffled construct has the highest potential to induce a strong E6- and E7-specific cytotoxic T lymphocytes as measured in different *in vitro* assays.

### *In vivo* tumor regression

**Figure 5:**
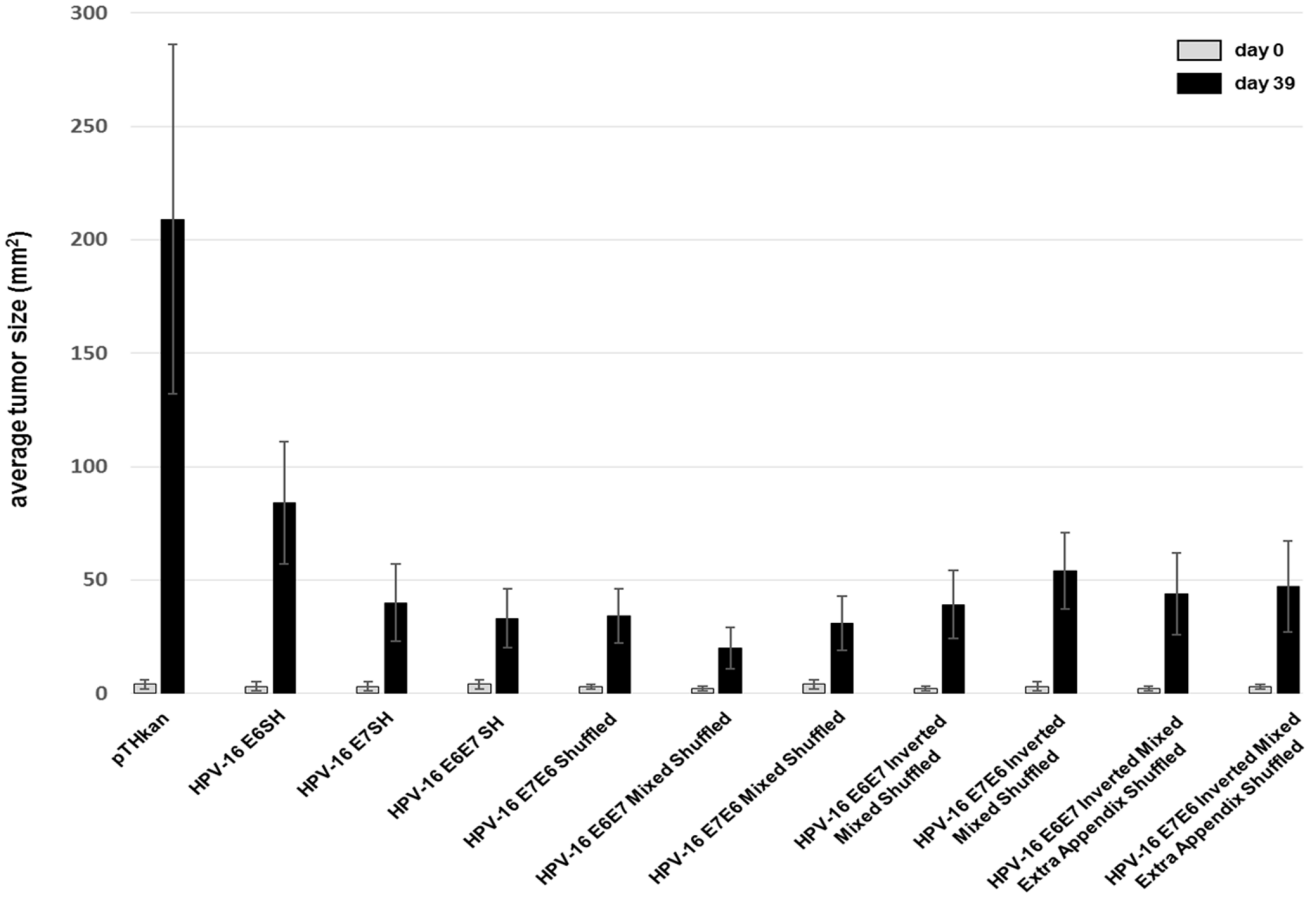
Growth of C3 tumors in mice after immunization with HPV-encoding vectors. Mice (n = 10/group) received tumor cells and were immunized twice with DNA (empty vector pTHkan, HPV-encoding vector plasmids as indicated) when the tumors were clearly palpable and surface tumor sizes were measured over time. Data gives the average tumor sizes at day 0 (day of first immunization) and at day 39 when the experiment was terminated ± SD, respectively. One of two experiment yielding very similar results is shown.

To verify the *in vitro* data in an HPV-specific tumor model we performed tumor regression experiments using HPV-16 E6 and E7 positive C3 cells in C57/B6 mice [22]. When small tumors in all animals were clearly palpable (designated day 0) they were immunized twice with 100 µg of DNA (1 mg/ml) as described in the Materials and Methods section. The quantification of the cellular immune responses in both Elispot assays as well as in the ^51^Cr-release assay correlated with the tumor regression. Indeed, the injection of theHPV-16 E6E7 mixed shuffled vaccine provoked the most effective control of the tumor outgrowth. At the end of the experiment, the average tumor size in the control group (empty vector) was 209±77 mm^2^ but only 20±9 mm^2^ in the E6E7 mixed shuffled treated group (p<0.0001) (figure 5). Additionally, only in the E6E7 mixed shuffled group one animal was completely tumor free. The second strongest HPV gene in this assay was the E7E6 mixed shuffled construct and the therapeutic effect in comparison to the E6E7 mixed shuffled gene was significantly reduced (p = 0.03).

## Discussion

Cervical cancer is the second most common cancer in women worldwide resulting in about half a million newly diagnosed cases and 260,000 deaths every year [27]. HPV 16, the prototype of high-risk papillomaviruses, is the principal cause of cervical cancer and its precursor lesions (high grade cervical intraepithelial neoplasia; CIN3), being responsible for more than half of the reported cases [28]. Studies established the role of the E6 and E7 oncoproteins in cell transformation through interaction with the p53 and pRB105 tumor suppressor proteins respectively and consequent progression of cell cycle to S phase and prevention of apoptosis [29]. Since both proteins are only expressed intracellularly, cell-mediated immunity controls growth and progression of HPV 16 induced lesions [30], [31]. Different E6 and/or E7 based vaccine preparations including purified proteins, synthetic peptides, recombinant vaccinia viruses and virus-like particles were evaluated for their efficacy in animal experiments and initial clinical trials [13], [32], [33], [34]. The results indicated that E6 and E7 proteins can be considered as promising targets for immunotherapeutic approaches against HPV-induced tumors.

Since the first description in the early 1990s, DNA vaccines proved to be a logical alternative to conventional protein-based vaccines. DNA vaccines stand out by their: 1) simple design and ease of production, 2) remarkable stability and long shelf-life, 3) considerable safety in clinical trials, 4) cost-effectiveness, and 5) ability to activate both arms of specific immunity [35]. The previous use of HPV 16 E7 in DNA vaccination was insufficient to induce effective CTL response [36], however, fusion with either a heat shock protein [37] or protein export signal [38] significantly improved its immunogenicity. The major concern of using E6 and E7 genes in a DNA vaccine lies in their transforming property, which is considered as risk for human recipients. The most common approach to abolish the transformation property of E6 and E7 involved mutating the active sites that mediate binding with p53 and pRB proteins [39], [40].

In our previous work we choose another strategy, i.e. the generation of rearranged genes through disruption of the transformation active sites and reassembly of gene fragments in a random fashion. To preclude the loss of potential CTL epitopes as a result of gene shuffling, an appendix that includes the original sequences at the junctions was added at the end of the constructs. The potential of this technology was first evaluated using shuffled HPV 16 E7 gene (HPV 16 E7SH) in murine fibroblast cells and C57BL/6 mice. The results indicated that E7SH was able to induce CTL response against the authentic protein with complete loss of the transformation activity [15]. Two approaches were employed to improve the immunogenicity and safety of such constructs. First we added a Kozak sequence 5′ to HPV 16 E7SH, codon-optimized the core –but not in the appendix– sequence for use in mammalian cells, and utilized the pTHamp vector, which is applicable for humans. This shuffled construct of second generation was able to induce high level of specific immune response as evaluated both *in vitro* and *in vivo* with no potential of oncogenicity [14]. Later we utilized the shuffled versions of E6 and E7 genes after fusion with the gene sequence that encodes Tetanus Toxin Fragment C domain 1 (TTFC). Preclinical safety and potency studies showed that both constructs are capable to provoke strong immune response with no risk of cell transformation [26], [41].

In the current study, we extended our strategy to combine E6 and E7 shuffled genes in a single construct aiming to provide the vaccine recipient with broader and more efficient immune repertoire. Eight different combinations of HPV 16 E6/E7 shuffled genes were designed with inclusion of 5′ Kozak sequence and codon optimization (figure 1). Furthermore, two sequences originated from the simian virus 40 (SV40) were introduced in E6/E7 SH gene constructs; a) SV40 J-domain, which was added next to Kozak sequence and is thought to increase the immunogenicity of chimeric antigens via its ability to bind heat shock proteins [42], b) SV40 translocation sequence, which was added at the 3′ end of the construct prior to stop codon. This sequence was reported to increase the expression and immunogenicity of plasmid-encoded proteins through enhanced nuclear transport [43]. All constructs were cloned in pTHkan vector instead of pTHamp, which is not recommended for use in humans due to the potential risk for the development of anaphylactic shock in susceptible individuals and generation of ampicillin-resistant strains of commensal bacteria [44].

In a first round of experiments we analyzed E6 and E7-specific IFN-gamma and Granzyme B responses in Elispot assays of immunized animals. All vaccine candidates investigated were able to induce specific responses, but interestingly one out of the eight constructs (E6E7 Mixed Shuffled) displayed a significant stronger immunogenic potential. This finding could be reproduced in the functional ^51^Cr-release assay and, most importantly, by *in vivo* tumor regression experiments. The aim of the study was the immunological characterization of newly developed E6 and E7-based therapeutic HPV-16 vaccine candidates rather than to investigate the mechanism(s) driving the different responses. Therefore, we can only speculate that changes in the nucleotide sequences may influence RNA abundance (either by synthesis or degradation) and therefore the amount of the encoded antigen. Indeed, it is well known that interactions between cis-regulatory sequences and different RNA-binding proteins as well as microRNAs influence RNA stability [45]. Another possible explanation for the clearly enhanced immunogenicity of one shuffled HPV construct could be modified protein stability. For example the nature of N-terminal residues influences the conjugation of ubiquitin which is a prerequisite for MHC class I presentation [46]. Additionally, it is known that the proteasome preferentially hydrolyzes peptides with defined amino acid stretches. As an example, the so called “peptidyl postglutamyl cleaving activity” cuts after aspartyl residues 30 times faster than after glutamates [47], which could positively influence epitope availability to MHC I molecules.

Together, the outcome of this study clearly demonstrates that gene shuffling is a potential approach to increase the immunogenicity of antigens. This finding may generally influence the development of DNA- and protein-based vaccine in general.

## Supporting Information

Materials and Methods S1 **Sequence of the eight different versions of the HPV 16 E6/E7 shuffled genes.**
(PDF)
