# Supplementary material for: Design of a Highly Effective Therapeutic HPV16 E6/E7-Specific DNA Vaccine: Optimization by Different Ways of Sequence Rearrangements (Shuffling)
Source: PLoS One. 2014 Nov 25;9(11):e113461. doi: 10.1371/journal.pone.0113461 (PMC4244082; doi:10.1371/journal.pone.0113461)
Supplement: Materials and Methods S1 — Sequence of the eight different versions of the HPV 16 E6/E7 shuffled genes. (PDF) [file pone.0113461.s001.pdf]

**SUPPLEMENTARY MATERIALS AND METHODS**

**Sequence of the eight different versions of the HPV E6/E7 shuffled genes (the Kozak sequence is underlined)**

**HPV 16 E6E7 Shuffled:**

5`-CCCGCCGCCACCATGGATAAAGTGCTGAACCGCGAAGAAAGCCTGCAGCTGATG
GATCTGCTGGGCCTGGAACGCAGCGCGTGGGGCAACATTCCGCTGATGCGCAAAGC
GTATCTGAAAAAATGCAAAGAATTTCATCCGGATAAAGGCGGCGATGAAGAAAAAA
TGAAAAAAATGAACACCCTGTATAAAAAAATGGAAGATGGCGTGAAATATGCGCAT
CAGCCGGATTTTGGCGGCTTTATGCATCAAAAGAGAACTGCAATGTTCCAGGACCCC
CAGGAGCGGCCCCGGAAGCTGCCCCAGCTGTGCACCGAGCTGCAGACCACCATCCA
CGACATCATCCTGGAGTGCGTGAACTGCCAGAAGCCCCTGTGCCCCGAGGAGAAGC
AGCGGCACCTGGACAAGAAGCAGCGGTTCCACAACATCCGGGGCCGGTGGACCGGC
CGGTGCATGAAGTGCCTGAAGTTCTACAGCAAGATCAGCGAGTACCGGCACTACTG
CTACAGCTTGTACGGCACCACCCTGGAGCAGCAGTACAACAAGCCCCTGTGCGACC
TGCTGATCCGGTGCATCTACTGCAAGCAGCAGCTGCTGCGGCGGGAGGTGTACGACT
TCGCCTTCCGGGACCTGTGCATCGTGTACCGGGACGGCAACCCCTACGCCGTGTGCG
ACAGCTGCTGCCGGAGCAGCCGGACCCGGCGGGAGACCCAGCTGATTCATGATATT
ATTCTTGAATGTGTTTATTGTAAACAACAACTTCTTCGTCGTGAAGATGGTAATCCTT
ATGCTGTTTGTGATAAATGTCTTAAATTTTATTCTAAAATTCTTTGTGATCTTCTTATT
CGTTGTATTAATTGTCAAAAACCTCTTTGTCCTGAACGTGGTCGTTGGACTGGTCGTT
GTATGTCTTGTTGTCGTTCTTCTCGTACTCGTATGCACGGCGACACCCCCACCCTGCA
CGAGTACATGCTGGACCTGCAGCCCGAGACCACCGACCTGTACTGCATCTGCAGCC
AGAAACCCAAGTGCGACAGCACCCTGCGGCTGTGCGTGCAGAGCACCCACGTGGAC

ATCCGGACCCTGGAGGACCTGCTGATGGGCACCCTGGGCATCGTGTGCCCCTACGA

GCAGCTGAACGACAGCAGCGAGGAGGAGGATGAGATCGACGGCCCCGCCGGCCAG

GCTGAGCCCGACCGGGCCCACTACAACATCGTGACCTTCTGCTGCCAACCAGAGAC

AACTGATCTCTACTGTTATGAGCAATTAAATGACAGCTCAGAGCATTACAATATTGT

AACCTTTTGTTGCAAGTGTGACTCTACGCTTCGGTTGTGCATGGGCACACTAGGAAT

TGTGTGCCCCATCTGTTCTCAGAAACCAACCAACGACTGATTAACTCTACGTACGAA

ACGTATGAAGACGGACGACCCCTCGGACCCCTGAAAGGTGTGGTAA-3`

**HPV 16 E7E6 Shuffled:**

5`-<u>CCCGCCGCCACC</u>ATGGATAAAGTGCTGAACCGCGAAGAAAGCCTGCAGCTGATG

GATCTGCTGGGCCTGGAACGCAGCGCGTGGGGCAACATTCCGCTGATGCGCAAAGC

GTATCTGAAAAAATGCAAAGAATTTCATCCGGATAAAGGCGGCGATGAAGAAAAAA

TGAAAAAAATGAACACCCTGTATAAAAAAATGGAAGATGGCGTGAAATATGCGCAT

CAGCCGGATTTTGGCGGCTTTATGCACGGCGACACCCCCACCCTGCACGAGTACATG

CTGGACCTGCAGCCCGAGACCACCGACCTGTACTGCATCTGCAGCCAGAAACCCAA

GTGCGACAGCACCCTGCGGCTGTGCGTGCAGAGCACCCACGTGGACATCCGGACCC

TGGAGGACCTGCTGATGGGCACCCTGGGCATCGTGTGCCCCTACGAGCAGCTGAAC

GACAGCAGCGAGGAGGAGGATGAGATCGACGGCCCCGCCGGCCAGGCTGAGCCCG

ACCGGGCCCACTACAACATCGTGACCTTCTGCTGCCAACCAGAGACAACTGATCTCT

ACTGTTATGAGCAATTAAATGACAGCTCAGAGCATTACAATATTGTAACCTTTTGTT

GCAAGTGTGACTCTACGCTTCGGTTGTGCATGGGCACACTAGGAATTGTGTGCCCCA

TCTGTTCTCAGAAACCAATGCATCAAAAGAGAACTGCAATGTTCCAGGACCCCCAG

GAGCGGCCCCGGAAGCTGCCCCAGCTGTGCACCGAGCTGCAGACCACCATCCACGA

CATCATCCTGGAGTGCGTGAACTGCCAGAAGCCCCTGTGCCCCGAGGAGAAGCAGC

GGCACCTGGACAAGAAGCAGCGGTTCCACAACATCCGGGGCCGGTGGACCGGCCGG

TGCATGAAGTGCCTGAAGTTCTACAGCAAGATCAGCGAGTACCGGCACTACTGCTAC

AGCTTGTACGGCACCACCCTGGAGCAGCAGTACAACAAGCCCCTGTGCGACCTGCT

GATCCGGTGCATCTACTGCAAGCAGCAGCTGCTGCGGCGGGAGGTGTACGACTTCG

CCTTCCGGGACCTGTGCATCGTGTACCGGGACGGCAACCCCTACGCCGTGTGCGACA

GCTGCTGCCGGAGCAGCCGGACCCGGCGGGAGACCCAGCTGATTCATGATATTATT

CTTGAATGTGTTTATTGTAAACAACAACTTCTTCGTCGTGAAGATGGTAATCCTTATG

CTGTTTGTGATAAATGTCTTAAATTTTATTCTAAAATTCTTTGTGATCTTCTTATTCGT

TGTATTAATTGTCAAAAACCTCTTTGTCCTGAACGTGGTCGTTGGACTGGTCGTTGTA

TGTCTTGTTGTCGTTCTTCTCGTACTCGTACCAACGACTGATTAACTCTACGTACGAA

ACGTATGAAGACGGACGACCCCTCGGACCCCTGAAAGGTGTGGTAA-3`

**HPV 16 E6E7 Mixed Shuffled:**

5`-<u>CCCGCCGCCACC</u>ATGGATAAAGTGCTGAACCGCGAAGAAAGCCTGCAGCTGATG

GATCTGCTGGGCCTGGAACGCAGCGCGTGGGGCAACATTCCGCTGATGCGCAAAGC

GTATCTGAAAAAATGCAAAGAATTTCATCCGGATAAAGGCGGCGATGAAGAAAAAA

TGAAAAAAATGAACACCCTGTATAAAAAAATGGAAGATGGCGTGAAATATGCGCAT

CAGCCGGATTTTGGCGGCTTTATGCATCAAAAGAGAACTGCAATGTTCCAGGACCCC

CAGGAGCGGCCCCGGAAGCTGCCCCAGCTGTGCACCGAGCTGCAGACCACCATCCA

CGACATCATCCTGGAGTGCGTGAACTGCCAGAAGCCCCTGTGCCCCGAGGAGAAGC

AGCGGCACCTGGACAAGAAGCAGCGGTTCCACAACATCCGGGGCCGGTGGACCGGC

CGGTGCATGAAGTGCCTGAAGTTCTACAGCAAGATCAGCGAGTACCGGCACTACTG

CTACAGCTTGTACGGCACCACCCTGGAGCAGCAGTACAACAAGCCCCTGTGCGACC

TGCTGATCCGGTGCATCATGCACGGCGACACCCCCACCCTGCACGAGTACATGCTGG

ACCTGCAGCCCGAGACCACCGACCTGTACTGCATCTGCAGCCAGAAACCCAAGTGC

GACAGCACCCTGCGGCTGTGCGTGCAGAGCACCCACGTGGACATCCGGACCCTGGA

GGACCTGCTGATGGGCACCCTGGGCATCGTGTGCCCCTACTGCAAGCAGCAGCTGCT

GCGGCGGGAGGTGTACGACTTCGCCTTCCGGGACCTGTGCATCGTGTACCGGGACG

GCAACCCCTACGCCGTGTGCGACAGCTGCTGCCGGAGCAGCCGGACCCGGCGGGAG

ACCCAGCTGATTCATGATATTATTCTTGAATGTGTTTATTGTAAACAACAACTTCTTC

GTCGTGAAGATGGTAATCCTTATGCTGTTTGTGATAAATGTCTTAAATTTTATTCTAA

AATTCTTTGTGATCTTCTTATTCGTTGTATTAATTGTCAAAAACCTCTTTGTCCTGAAC

GTGGTCGTTGGACTGGTCGTTGTATGTCTTGTTGTCGTTCTTCTCGTACTCGTTACGA

GCAGCTGAACGACAGCAGCGAGGAGGAGGATGAGATCGACGGCCCCGCCGGCCAG

GCTGAGCCCGACCGGGCCCACTACAACATCGTGACCTTCTGCTGCCAACCAGAGAC

AACTGATCTCTACTGTTATGAGCAATTAAATGACAGCTCAGAGCATTACAATATTGT

AACCTTTTGTTGCAAGTGTGACTCTACGCTTCGGTTGTGCATGGGCACACTAGGAAT

TGTGTGCCCCATCTGTTCTCAGAAACCAACCAACGACTGATTAACTCTACGTACGAA

ACGTATGAAGACGGACGACCCCTCGGACCCCTGAAAGGTGTGGTAA-3`

**HPV 16 E7E6 Mixed Shuffled:**

5`-CCCGCCGCCACCATGGATAAAGTGCTGAACCGCGAAGAAAGCCTGCAGCTGATG

GATCTGCTGGGCCTGGAACGCAGCGCGTGGGCAACATTCCGCTGATGCGCAAAGCGTAT

CTGAAAAAATGCAAAGAATTTCATCCGGATAAAGGCGGCGATGAAGAAAAAATGA

AAAAAATGAACACCCTGTATAAAAAAATGGAAGATGGCGTGAAATATGCGCATCAG

CCGGATTTTGGCGGCTTTATGCACGGCGACACCCCCACCCTGCACGAGTACATGCTG

GACCTGCAGCCCGAGACCACCGACCTGTACTGCATCTGCAGCCAGAAACCCAAGTG

CGACAGCACCCTGCGGCTGTGCGTGCAGAGCACCCACGTGGACATCCGGACCCTGG

AGGACCTGCTGATGGGCACCCTGGGCATCGTGTGCCCCATGCATCAAAAGAGAACT

GCAATGTTCCAGGACCCCCAGGAGCGGCCCCGGAAGCTGCCCCAGCTGTGCACCGA

GCTGCAGACCACCATCCACGACATCATCCTGGAGTGCGTGAACTGCCAGAAGCCCC

TGTGCCCCGAGGAGAAGCAGCGGCACCTGGACAAGAAGCAGCGGTTCCACAACATC

CGGGGCCGGTGGACCGGCCGGTGCATGAAGTGCCTGAAGTTCTACAGCAAGATCAG

CGAGTACCGGCACTACTGCTACAGCTTGTACGGCACCACCCTGGAGCAGCAGTACA

ACAAGCCCCTGTGCGACCTGCTGATCCGGTGCATCTACGAGCAGCTGAACGACAGC

AGCGAGGAGGAGGATGAGATCGACGGCCCCGCCGGCCAGGCTGAGCCCGACCGGG

CCCACTACAACATCGTGACCTTCTGCTGCCAACCAGAGACAACTGATCTCTACTGTT

ATGAGCAATTAAATGACAGCTCAGAGCATTACAATATTGTAACCTTTTGTTGCAAGT

GTGACTCTACGCTTCGGTTGTGCATGGGCACACTAGGAATTGTGTGCCCCATCTGTT

CTCAGAAACCATACTGCAAGCAGCAGCTGCTGCGGCGGGAGGTGTACGACTTCGCC

TTCCGGGACCTGTGCATCGTGTACCGGGACGGCAACCCCTACGCCGTGTGCGACAGC

TGCTGCCGGAGCAGCCGGACCCGGCGGGAGACCCAGCTGATTCATGATATTATTCTT

GAATGTGTTTATTGTAAACAACAACTTCTTCGTCGTGAAGATGGTAATCCTTATGCT

GTTTGTGATAAATGTCTTAAATTTTATTCTAAAATTCTTTGTGATCTTCTTATTCGTTG

TATTAATTGTCAAAAACCTCTTTGTCCTGAACGTGGTCGTTGGACTGGTCGTTGTATG

TCTTGTTGTCGTTCTTCTCGTACTCGTACCAACGACTGATTAACTCTACGTACGAAAC

GTATGAAGACGGACGACCCCTCGGACCCCTGAAAGGTGTGGTAA-3`

**HPV 16 E6E7 Inverted Mixed Shuffled:**

5`-CCCGCCGCCACCATGGATAAAGTGCTGAACCGCGAAGAAAGCCTGCAGCTGATG
GATCTGCTGGGCCTGGAACGCAGCGCGTGGGGCAACATTCCGCTGATGCGCAAAGC
GTATCTGAAAAAATGCAAAGAATTTCATCCGGATAAAGGCGGCGATGAAGAAAAAA
TGAAAAAAATGAACACCCTGTATAAAAAAATGGAAGATGGCGTGAAATATGCGCAT
CAGCCGGATTTTGGCGGCTTTTACTGCAAGCAGCAGCTGCTGCGGCGGGAGGTGTAC
GACTTCGCCTTCCGGGACCTGTGCATCGTGTACCGGGACGGCAACCCCTACGCCGTG
TGCGACAGCTGCTGCCGGAGCAGCCGGACCCGGCGGGAGACCCAGCTGATTCATGA
TATTATTCTTGAATGTGTTTATTGTAAACAACAACTTCTTCGTCGTGAAGATGGTAAT
CCTTATGCTGTTTGTGATAAATGTCTTAAATTTTATTCTAAAATTCTTTGTGATCTTCT
TATTCGTTGTATTAATTGTCAAAAACCTCTTTGTCCTGAACGTGGTCGTTGGACTGGT
CGTTGTATGTCTTGTTGTCGTTCTTCTCGTACTCGTTACGAGCAGCTGAACGACAGCA
GCGAGGAGGAGGATGAGATCGACGGCCCCGCCGGCCAGGCTGAGCCCGACCGGGC
CCACTACAACATCGTGACCTTCTGCTGCCAACCAGAGACAACTGATCTCTACTGTTA
TGAGCAATTAAATGACAGCTCAGAGCATTACAATATTGTAACCTTTTGTTGCAAGTG
TGACTCTACGCTTCGGTTGTGCATGGGCACACTAGGAATTGTGTGCCCCATCTGTTCT
CAGAAACCAATGCATCAAAAGAGAACTGCAATGTTCCAGGACCCCCAGGAGCGGCC
CCGGAAGCTGCCCCAGCTGTGCACCGAGCTGCAGACCACCATCCACGACATCATCCT
GGAGTGCGTGAACTGCCAGAAGCCCCTGTGCCCCGAGGAGAAGCAGCGGCACCTGG
ACAAGAAGCAGCGGTTCCACAACATCCGGGGCCGGTGGACCGGCCGGTGCATGAAG
TGCCTGAAGTTCTACAGCAAGATCAGCGAGTACCGGCACTACTGCTACAGCTTGTAC
GGCACCACCCTGGAGCAGCAGTACAACAAGCCCCTGTGCGACCTGCTGATCCGGTG
CATCATGCACGGCGACACCCCCACCCTGCACGAGTACATGCTGGACCTGCAGCCCG

AGACCACCGACCTGTACTGCATCTGCAGCCAGAAACCCAAGTGCGACAGCACCCTG

CGGCTGTGCGTGCAGAGCACCCACGTGGACATCCGGACCCTGGAGGACCTGCTGAT

GGGCACCCTGGGCATCGTGTGCCCCACCAACGACTGATTAACTCTACGTACGAAAC

GTATGAAGACGGACGACCCCTCGGACCCCTGAAAGGTGTGGTAA-3`

**HPV 16 E7E6 Inverted Mixed Shuffled:**

5`-CCCGCCGCCACCATGGATAAAGTGCTGAACCGCGAAGAAAGCCTGCAGCTGATG

GATCTGCTGGGCCTGGAACGCAGCGCGTGGGGCAACATTCCGCTGATGCGCAAAGC

GTATCTGAAAAAATGCAAAGAATTTCATCCGGATAAAGGCGGCGATGAAGAAAAAA

TGAAAAAAATGAACACCCTGTATAAAAAAATGGAAGATGGCGTGAAATATGCGCAT

CAGCCGGATTTTGGCGGCTTTTACGAGCAGCTGAACGACAGCAGCGAGGAGGAGGA

TGAGATCGACGGCCCCGCCGGCCAGGCTGAGCCCGACCGGGCCCACTACAACATCG

TGACCTTCTGCTGCCAACCAGAGACAACTGATCTCTACTGTTATGAGCAATTAAATG

ACAGCTCAGAGCATTACAATATTGTAACCTTTTGTTGCAAGTGTGACTCTACGCTTC

GGTTGTGCATGGGCACACTAGGAATTGTGTGCCCCATCTGTTCTCAGAAACCATACT

GCAAGCAGCAGCTGCTGCGGCGGGAGGTGTACGACTTCGCCTTCCGGGACCTGTGC

ATCGTGTACCGGGACGGCAACCCCTACGCCGTGTGCGACAGCTGCTGCCGGAGCAG

CCGGACCCGGCGGGAGACCCAGCTGATTCATGATATTATTCTTGAATGTGTTTATTG

TAAACAACAACTTCTTCGTCGTGAAGATGGTAATCCTTATGCTGTTTGTGATAAATG

TCTTAAATTTTATTCTAAAATTCTTTGTGATCTTCTTATTCGTTGTATTAATTGTCAAA

AACCTCTTTGTCCTGAACGTGGTCGTTGGACTGGTCGTTGTATGTCTTGTTGTCGTTC

TTCTCGTACTCGTATGCACGGCGACACCCCCACCCTGCACGAGTACATGCTGGACCT

GCAGCCCGAGACCACCGACCTGTACTGCATCTGCAGCCAGAAACCCAAGTGCGACA

GCACCCTGCGGCTGTGCGTGCAGAGCACCCACGTGGACATCCGGACCCTGGAGGAC

CTGCTGATGGGCACCCTGGGCATCGTGTGCCCCATGCATCAAAAGAGAACTGCAAT

GTTCCAGGACCCCCAGGAGCGGCCCCGGAAGCTGCCCCAGCTGTGCACCGAGCTGC

AGACCACCATCCACGACATCATCCTGGAGTGCGTGAACTGCCAGAAGCCCCTGTGC

CCCGAGGAGAAGCAGCGGCACCTGGACAAGAAGCAGCGGTTCCACAACATCCGGG

GCCGGTGGACCGGCCGGTGCATGAAGTGCCTGAAGTTCTACAGCAAGATCAGCGAG

TACCGGCACTACTGCTACAGCTTGTACGGCACCACCCTGGAGCAGCAGTACAACAA

GCCCCTGTGCGACCTGCTGATCCGGTGCATCACCAACGACTGATTAACTCTACGTAC

GAAACGTATGAAGACGGACGACCCCTCGGACCCCTGAAAGGTGTGGTAA-3`

**HPV 16 E6E7 Inverted Mixed Extra-Appendix Shuffled:**

5`-CCCGCCGCCACCATGGATAAAGTGCTGAACCGCGAAGAAAGCCTGCAGCTGATG

GATCTGCTGGGCCTGGAACGCAGCGCGTGGGGCAACATTCCGCTGATGCGCAAAGC

GTATCTGAAAAAATGCAAAGAATTTCATCCGGATAAAGGCGGCGATGAAGAAAAAA

TGAAAAAAATGAACACCCTGTATAAAAAAATGGAAGATGGCGTGAAATATGCGCAT

CAGCCGGATTTTGGCGGCTTTTACTGCAAGCAGCAGCTGCTGCGGCGGGAGGTGTAC

GACTTCGCCTTCCGGGACCTGTGCATCGTGTACCGGGACGGCAACCCCTACGCCGTG

TGCGACAGCTGCTGCCGGAGCAGCCGGACCCGGCGGGAGACCCAGCTGTACGAGCA

GCTGAACGACAGCAGCGAGGAGGAGGATGAGATCGACGGCCCCGCCGGCCAGGCT

GAGCCCGACCGGGCCCACTACAACATCGTGACCTTCTGCTGCATGCATCAAAAGAG

AACTGCAATGTTCCAGGACCCCCAGGAGCGGCCCCGGAAGCTGCCCCAGCTGTGCA

CCGAGCTGCAGACCACCATCCACGACATCATCCTGGAGTGCGTGAACTGCCAGAAG

CCCCTGTGCCCCGAGGAGAAGCAGCGGCACCTGGACAAGAAGCAGCGGTTCCACAA

CATCCGGGGCCGGTGGACCGGCCGGTGCATGAAGTGCCTGAAGTTCTACAGCAAGA

TCAGCGAGTACCGGCACTACTGCTACAGCTTGTACGGCACCACCCTGGAGCAGCAG

TACAACAAGCCCCTGTGCGACCTGCTGATCCGGTGCATCATGCACGGCGACACCCCC

ACCCTGCACGAGTACATGCTGGACCTGCAGCCCGAGACCACCGACCTGTACTGCATC

TGCAGCCAGAAACCCAAGTGCGACAGCACCCTGCGGCTGTGCGTGCAGAGCACCCA

CGTGGACATCCGGACCCTGGAGGACCTGCTGATGGGCACCCTGGGCATCGTGTGCC

CCATTCATGATATTATTCTTGAATGTGTTTATTGTAAACAACAACTTCTTCGTCGTGA

AGATGGTAATCCTTATGCTGTTTGTGATAAATGTCTTAAATTTTATTCTAAAATTCTT

TGTGATCTTCTTATTCGTTGTATTAATTGTCAAAAACCTCTTTGTCCTGAACGTGGTC

GTTGGACTGGTCGTTGTATGTCTTGTTGTCGTTCTTCTCGTACTCGTCAACCAGAGAC

AACTGATCTCTACTGTTATGAGCAATTAAATGACAGCTCAGAGCATTACAATATTGT

AACCTTTTGTTGCAAGTGTGACTCTACGCTTCGGTTGTGCATGGGCACACTAGGAAT

TGTGTGCCCCATCTGTTCTCAGAAACCAACCAACGACTGATTAACTCTACGTACGAA

ACGTATGAAGACGGACGACCCCTCGGACCCCTGAAAGGTGTGGTAA-3`

**HPV 16 E7E6 Inverted Mixed Extra-Appendix Shuffled:**

5`-CCCGCCGCCACCATGGATAAAGTGCTGAACCGCGAAGAAAGCCTGCAGCTGATG

GATCTGCTGGGCCTGGAACGCAGCGCGTGGGGCAACATTCCGCTGATGCGCAAAGC

GTATCTGAAAAAATGCAAAGAATTTCATCCGGATAAAGGCGGCGATGAAGAAAAAA

TGAAAAAAATGAACACCCTGTATAAAAAAATGGAAGATGGCGTGAAATATGCGCAT

CAGCCGGATTTTGGCGGCTTTTACGAGCAGCTGAACGACAGCAGCGAGGAGGAGGA

TGAGATCGACGGCCCCGCCGGCCAGGCTGAGCCCGACCGGGCCCACTACAACATCG

TGACCTTCTGCTGCTACTGCAAGCAGCAGCTGCTGCGGCGGGAGGTGTACGACTTCG

CCTTCCGGGACCTGTGCATCGTGTACCGGGACGGCAACCCCTACGCCGTGTGCGACA

GCTGCTGCCGGAGCAGCCGGACCCGGCGGGAGACCCAGCTGATGCACGGCGACACC

CCCACCCTGCACGAGTACATGCTGGACCTGCAGCCCGAGACCACCGACCTGTACTGC

ATCTGCAGCCAGAAACCCAAGTGCGACAGCACCCTGCGGCTGTGCGTGCAGAGCAC

CCACGTGGACATCCGGACCCTGGAGGACCTGCTGATGGGCACCCTGGGCATCGTGT

GCCCCATGCATCAAAAGAGAACTGCAATGTTCCAGGACCCCCAGGAGCGGCCCCGG

AAGCTGCCCCAGCTGTGCACCGAGCTGCAGACCACCATCCACGACATCATCCTGGA

GTGCGTGAACTGCCAGAAGCCCCTGTGCCCCGAGGAGAAGCAGCGGCACCTGGACA

AGAAGCAGCGGTTCCACAACATCCGGGGCCGGTGGACCGGCCGGTGCATGAAGTGC

CTGAAGTTCTACAGCAAGATCAGCGAGTACCGGCACTACTGCTACAGCTTGTACGGC

ACCACCCTGGAGCAGCAGTACAACAAGCCCCTGTGCGACCTGCTGATCCGGTGCAT

CCAACCAGAGACAACTGATCTCTACTGTTATGAGCAATTAAATGACAGCTCAGAGC

ATTACAATATTGTAACCTTTTGTTGCAAGTGTGACTCTACGCTTCGGTTGTGCATGGG

CACACTAGGAATTGTGTGCCCCATCTGTTCTCAGAAACCAATTCATGATATTATTCTT

GAATGTGTTTATTGTAAACAACAACTTCTTCGTCGTGAAGATGGTAATCCTTATGCT

GTTTGTGATAAATGTCTTAAATTTTATTCTAAAATTCTTTGTGATCTTCTTATTCGTTG

TATTAATTGTCAAAAACCTCTTTGTCCTGAACGTGGTCGTTGGACTGGTCGTTGTATG

TCTTGTTGTCGTTCTTCTCGTACTCGTACCAACGACTGATTAACTCTACGTACGAAAC

GTATGAAGACGGACGACCCCTCGGACCCCTGAAAGGTGTGGTAA-3`
